# Erythromycin resistance of clinical *Campylobacter jejuni* and *Campylobacter coli* in Shanghai, China

**DOI:** 10.3389/fmicb.2023.1145581

**Published:** 2023-05-16

**Authors:** Fen Gao, Lihong Tu, Mingliang Chen, Hongyou Chen, Xi Zhang, Yuan Zhuang, Jiayuan Luo, Min Chen

**Affiliations:** ^1^Department of Microbiology, Shanghai Municipal Center for Disease Control and Prevention, Shanghai, China; ^2^Department of Public Health Service and Safety Assessment, Shanghai Municipal Center for Disease Control and Prevention, Shanghai, China

**Keywords:** campylobacter, antimicrobial resistance, whole-genome sequencing, erythromycin resistance, *erm*(B)

## Abstract

*Campylobacter* species are zoonotic pathogens, as well as the prevalent cause of foodborne bacterial gastroenteritis. The spread of antimicrobial-resistant strains poses a serious threat to global public health and attracts attention worldwide, but information about clinical *Campylobacter* is relatively limited compared to isolates from food and animals. The current study illustrated the prevalence and antimicrobial resistance profiles of *Campylobacter jejuni* and *Campylobacter coli* isolates collected from a consecutive surveillance program between 2012 and 2019 in Shanghai, China, using antimicrobial susceptibility testing and whole-genome sequencing. Among the 891 *Campylobacter* strains (761 *C. jejuni* and 130 *C. coli*) isolates collected, high portions above 90% of resistance to ciprofloxacin, nalidixic acid, and tetracycline were observed for both *C. jejuni* and *C. coli*. The most common MDR profiles represented by *C. jejuni* and *C. coli* were combination of ciprofloxacin, tetracycline, florfenicol and nalidixic acid (5.39%), and azithromycin, ciprofloxacin, erythromycin, gentamicin, tetracycline, clindamycin, nalidixic acid (28.46%), respectively. The erythromycin resistance of *C. coli* (59.23%) is higher than *C. jejuni* (2.50%). A total of 76 erythromycin resistant isolates (16 *C. jejuni* and 60 *C. coli*) were sequenced using Illumina platform for determining the genotypes, antimicrobial resistance patterns and phylogeny analysis. Multilocus sequence typing (MLST) analysis showed a high genetic diversity with 47 sequence types (STs), including 4 novel alleles and 12 new STs. The most abundant clonal complexes (CCs) were CC-403 (31.25%) and CC-828 (88.33%) for *C. jejuni* and *C. coli*, respectively. Among the 76 erythromycin-resistant isolates, mutation A2075G in 23S rRNA and *erm*(B) gene were detected in 53.95 and 39.47%, respectively. The *erm*(B) gene was identified exclusively in 30 *C. coli* isolates. All these *erm*(B) positive isolates were multi-drug resistant. Furthermore, comparison of the *erm*(B)-carrying isolates of multiple sources worldwide demonstrated the possibility of zoonotic transmission of *erm*(B) in *Campylobacter*. These findings highlight the importance of continuous surveillance of erythromycin resistance dissemination in *Campylobacter* which may compromise the effectiveness of antimicrobial therapy.

## Introduction

*Campylobacter* species are among the most common foodborne bacterial pathogens causing acute gastroenteritis in human beings around the world, especially for *Campylobacter jejuni* and *Campylobacter coli* (Man, 2011). Campylobacteriosis is also the first most reported zoonosis in humans in the European Union (EU), and of the campylobacteriosis cases, 88.4% were *C. jejuni*, 10.1% were *C. coli* in 2021 with species information provided (Authority et al., 2022). It has been estimated that *Campylobacter* is responsible for around 9 million human infection cases each year and EUR 2.4 billion economic losses annually in the EU (EFSA, 2014). As a zoonotic and foodborne pathogen, *Campylobacter* can be transmitted to human through raw or undercooked chicken, unpasteurized milk, contaminated food and water, or contact with animals (Kaakoush et al., 2015).

*Campylobacter* infection usually causes acute, self-limited gastrointestinal illness manifested as diarrhea, fever, abdominal cramps, and even autoimmune illness known as Guillain–Barré syndrome (GBS) and Miller Fisher syndrome in rare severe cases (Griffiths and Park, 1990; Takahashi et al., 2005; Scallan et al., 2015). Clinical antimicrobial treatments for severe infection are necessary, with macrolides and fluoroquinolones being prescribed occasionally (Kaakoush et al., 2015). However, considering the occurrence and the rising of resistance to azithromycin and ciprofloxacin, drug-resistant *Campylobacter* was regarded as a serious public health threat in the United States by the Centers for Disease Control and Prevention (Solomon and Oliver, 2014). With the increasing prevalence of fluoroquinolone resistance worldwide, *Campylobacter* spp. has been recognized as one of the six pathogens in a list of antibiotic resistant bacteria with high priority all over the world by the World Health Organization (Tacconelli et al., 2017). Furthermore, the emergence and distribution of macrolide resistance among *Campylobacter* will also pose a threat for clinical treatment. Meanwhile, *Campylobacter* isolates from both developed and developing countries with increasing resistance to multiple antimicrobials, including aminoglycosides, fluoroquinolones, macrolides, and tetracyclines have been reported (Moore et al., 2006; Zhou et al., 2016), and the use of antibiotics in agriculture and clinical could contribute to progression of antimicrobial resistance (Wimalarathna et al., 2013), which necessitate the continuous surveillance of antimicrobial resistance, characterization of the underlying resistance determinants and antimicrobial resistance dissemination mechanism of *Campylobacter* isolates.

With the increasing awareness of the major public health importance of *Campylobacter*, investigations about the prevalence of *Campylobacter* isolated from clinical, livestock and poultry industry in China have been carried out in the recent years, accompanying with deepening investigation of the related genomic characterizations (Zhang et al., 2016; Zhou et al., 2016; Liu et al., 2019). Particularly, previous investigations revealed the high level macrolide resistance rate of *Campylobacter* isolates in China (Qin et al., 2011; Zhang et al., 2016), whereas the rate remained below 10% of some developed countries (Gibreel and Taylor, 2006). Macrolide resistance among *Campylobacter* species has been mainly attributed to mutations in target genes (23S rRNA and ribosomal proteins L4 and L22), CmeABC efflux system, or 23S rRNA methyltransferase coded by *erm*(B) gene (Bolinger and Kathariou, 2017). Among the reported three major mechanisms of macrolide resistance, the *erm*(B) mechanism first discovered in 2014 (Qin et al., 2014) is particular worrisome, due to the high-level resistance conferred to macrolides and horizontal transfer of *erm*(B) among *Campylobacter* along with other antibiotic resistance genes, which contribute to the spread of multiple resistance (Liu et al., 2019).

In Shanghai Municipality of China, a consecutive surveillance program of outpatients with diarrhea based on public health laboratories in Shanghai Municipal Center for Disease Control and Prevention (Shanghai CDC) and sentinel hospitals has been started since 2012. Consequently, we analyzed the antimicrobial resistance of clinical *Campylobacter* isolates from patients with diarrhea retrospectively in this study, evaluated the prevalence of erythromycin-resistant isolates and determined the molecular characteristics of the *erm*(B)-positive isolates from 2012 to 2019 using whole genome sequencing (WGS) data.

## Materials and methods

### Strain collection and culture conditions

As part of the pathogen surveillance program of outpatients with diarrhea, monitoring of *Campylobacter* spp. was started in 2012 in Shanghai. In this *Campylobacter* surveillance, fecal samples from diarrheal outpatients were collected and cultured for *Campylobacter* spp. isolation based on the filter-based method as described previously (Shiramaru et al., 2012) using *Campylobacter* isolation kit (ZC-CAMPY-001, Qingdao Sinova-HK Biotechnology Co., Ltd., Qingdao, China). In brief, the swab of fecal sample was suspended in *Campylobacter* enrichment broth and subsequently filtered through membrane filter (0.45 μm) onto Columbia blood agar containing 5% sheep blood for isolation. After removal of filters and incubation at 42°C for 24−48 h under microaerobic environment (5% O_2_, 10% CO_2_, and 85% N_2_), single colony was picked onto a new Columbia agar plate for incubation and followed by identification using the matrix-assisted laser desorption/ionization time of fight mass spectrometry (MALDI-TOF-MS, bioMérieux, France) and PCR analysis. A total of 891 non-duplicate *Campylobacter* spp. isolates were collected from 47,965 patients suffering from diarrhea consecutively during 2012–2019 in Shanghai. Meanwhile, the *Campylobacter* surveillance network gradually developed covering every public health laboratories in 16 Shanghai District CDC and 23 sentinel hospitals from only several pilot hospitals participated in the project in the beginning. All isolates were deposited at −80°C.

### Antimicrobial susceptibility testing

Minimum inhibitory concentrations (MICs) of all the *Campylobacter* isolates were determined using the broth microdilution method for eight antimicrobial agents of six classes, including aminoglycosides (gentamicin, GEN), macrolides (erythromycin, ERY and azithromycin, AZI), lincosamides (clindamycin, CLI), phenicols (florfenicol, FFN), quinolones (ciprofloxacin, CIP and nalidixic acid, NAL), tetracyclines (tetracycline, TET) with commercial kits (Shanghai Fosun Long March Medical Science Co., xLtd., Shanghai, China). MICs were interpreted in accordance with the standard of The National Antimicrobial Resistance Monitoring System (NARMS, www.cdc.gov/narms/antibiotics-tested.html). The cut-off points of resistance were set as ≥8 μg/ml for FFN, ≥1 μg/ml for CIP, ≥4 μg/ml for GEN, ≥32 μg/ml for NAL for both *C. jejuni* and *C. coli*. MIC breakpoints were set as ≥1 μg/ml for CLI, ≥8 μg/ml for ERY, ≥0.5 μg/ml for AZI, ≥2 μg/ml for TET for *C. jejuni*, and ≥ 2 μg/ml for CLI, ≥16 μg/ml for ERY, ≥1 μg/ml for AZI, ≥4 μg/ml for TET for *C. coli*, respectively. Quality control was performed with *C. jejuni* ATCC 33560.

### Genomic sequencing and phylogenetic analysis

Erythromycin-resistant isolates were selected for whole genome sequencing. For genomic DNA extraction, QIAamp DNA mini kit (Qiagen, Germany) was used according to the manufacturer’s instructions. Genomes were sequenced with Illumina Hiseq platform (Illumina, Inc., United States) using 150 bp paired-end protocol and WGS reads obtained have been submitted to the Sequence Read Archive database of the NCBI under accession numbers SRR22936162 to SRR22936237. The genomes were assembled *de novo* using SPAdes (Bankevich et al., 2012). Snippy 3.2 was used to identify core single-nucleotide polymorphisms (SNPs), and recombination regions were excluded using ClonalFrameML 1.12 (Didelot and Wilson, 2015). Based on the recombinant-free SNPs, the maximum likelihood phylogeny was built by using RAxML (Stamatakis, 2006; Stamatakis et al., 2008). The phylogenetic tree was visualized using iTOL (Letunic and Bork, 2007) associating antimicrobial resistance profile of each isolates.

### Multilocus sequence typing

Multilocus sequence typing (MLST) profiles were determined using online tools described previously (Jolley et al., 2018). Genomic sequences were queried against the *C. jejuni/coli* PubMLST database^1^ for identifying seven allele loci (*aspA*, *glnA*, *gltA*, *glyA*, *pgm*, *tkt*, and *uncA*), corresponding sequence type (ST) and clonal complex (cc). New alleles and ST profiles were assigned by submitting the sequences to the PubMLST database using the submission database. A minimum spanning tree (MST) was constructed based on the MLST data obtained in this work and from other unpublished studies previously carried out in our lab, using BioNumerics v.6.5 (bioMérieux, Marcy-l’Étoile, France).

### Analysis of resistance profile

Screening of contigs for antimicrobial resistance genes were performed using Abricate v.0.8^2^ and NCBI AMRFinderPlus^3^ (Feldgarden et al., 2019). Resistance gene hits were filtered according to a minimum DNA identity of 80% and sequence coverage of 70%. BLAST 2.6.0+ was used to detect point mutations in the 23S rRNA gene (nucleotides at position 2074 and 2075), *gyrA* gene (T → I at amino acid 86 in the encoded protein), and the presence of RE-*cmeABC* (a resistance-enhancing variant of *cmeABC*) associated with macrolide, quinolone and enhanced multidrug resistance, respectively.

### Statistical analysis

Statistical differences (*p* < 0.05) for the antimicrobial resistances of *Campylobacter* isolates were determined using IBM SPSS software, version 20.0 (IBM, Corp.).

## Results

### Susceptibility test of *Campylobacter jejuni* and *Campylobacter coli* isolates

A total of 891 *Campylobacter* strains comprising 761 *C. jejuni* (85.41%, 761/891) and 130 *C. coli* (14.59%, 130/891) isolates were collected between 2012 and 2019 (Table 1). Every single strain was obtained from one positive case of *Campylobacter* infection. *Campylobacter jejuni* was the predominate *Campylobacter* species recovered from clinical cases. As illustrated in Table 1, *C. jejuni* showed resistance most frequently to CIP (96.45%), followed by NAL (94.61%), TET (90.54%), CLI (16.56%), FFN (8.28%), GEN (6.57%), AZI (4.99%) and ERY (2.50%), while *C. coli* displayed highest resistance rate to CIP (100%), followed by NAL (98.46%), TET (96.15%), AZI (60.00%), ERY/GEN (59.23%), CLI (58.46%) and FFN (16.15%). Compared with *C. jejuni*, statistical higher resistant rates were observed in *C. coli* for GEN (*χ*^2^ = 251.929, *p* < 0.0001), CLI (*χ*^2^ = 111.213, *p* < 0.0001), ERY (*χ*^2^ = 371.753, *p* < 0.0001), AZI (*χ*^2^ = 296.671, *p* < 0.0001), FFN (*χ*^2^ = 8.065, *p* = 0.005) and TET (*χ*^2^ = 4.434, *p* = 0.035).

**Table 1:** Antimicrobial resistance of the isolates used in this study.

Antibiotic	2012–2016		2017		2018		2019		Total	
	*C. jejuni* (*n* = 237)	*C. coli* (*n* = 28)	*C. jejuni* (*n* = 106)	*C. coli* (*n* = 23)	*C. jejuni* (*n* = 162)	*C. coli* (*n* = 31)	*C. jejuni* (*n* = 256)	*C. coli* (*n* = 48)	*C. jejuni* (*n* = 761)	*C. coli* (*n* = 130)
CIP	94.51	100.00	95.28	100.00	96.91	100.00	98.44	100.00	96.45	100.00
NAL	88.19	92.86	95.28	100.00	96.91	100.00	98.83	100.00	94.61	98.46
TET	86.92	89.29	91.51	100.00	93.83	100.00	91.41	95.83	90.54	96.15
GEN	3.80	42.86	5.66	73.91	11.73	61.29	6.25	60.42	6.57	59.23
FFN	6.33	7.14	5.66	13.04	7.41	16.13	11.72	22.92	8.28	16.15
AZI	2.11	50.00	5.66	56.52	9.26	61.29	4.69	66.67	4.99	60.00
ERY	1.69	57.14	2.83	56.52	4.94	54.84	1.56	64.58	2.50	59.23
CLI	8.86	46.43	15.09	60.87	20.37	58.06	21.88	64.58	16.56	58.46

The most prevalent resistance profile for *C. jejuni* was CIP-TET-NAL (534/761, 70.17%), while two strains were resistant to all the eight antimicrobial agents tested (Table 2). The commonly MDR (multi-drug resistance) combination of *C. jejuni* was CIP-TET-FFN-NAL (41/761, 5.39%). For the case of *C. coli*, the prevalent resistance profile was AZI-CIP-ERY-GEN-TET-NAL-CLI (37/130, 28.46%), while 20 strains were resistant to all the eight antimicrobial agents (Table 2). It is noteworthy that the resistance to CIP, NAL, and TET were extremely high for both *C. jejuni* and *C. coli* isolates, with values exceeding 90% (Table 1). Such high levels of antimicrobial resistance rate indicated that these classes of antibiotics are probably unsuitable for empirical medication. Differences were observed in resistances to other antimicrobials between *C. jejuni* and *C. coli* isolates (Table 1). Specifically, erythromycin resistance in *C. coli* (59.23%) is much more prevalent than that in *C. jejuni* (2.50%). Of the 891 isolates analyzed, 121 *C. jejuni* (15.90%) and 96 *C. coli* (73.85%) isolates were multidrug-resistant, respectively. Two *C. jejuni* and 20 *C. coli* isolates were resistant to all the eight antimicrobials tested (Table 2).

**Table 2:** Antimicrobial resistance profiles detected among 891 *Campylobacter* isolates from Shanghai.

Resistance profile	*Campylobacter jejuni* isolates (%)	*Campylobacter coli* isolates (%)
AZI-CIP-ERY-GEN-TET-FEN-NAL-CLI	2 (0.26)	20 (15.38)
AZI-CIP-ERY-GEN-TET-NAL-CLI	6 (0.79)	37 (28.46)
AZI-CIP-ERY-TET-FEN-NAL-CLI	1 (0.13)	1 (0.77)
AZI-CIP-GEN-TET-FEN-NAL-CLI	1 (0.13)	0 (0)
AZI-CIP-ERY-GEN-NAL-CLI	0 (0)	1 (0.77)
AZI-CIP-ERY-GEN-TET-NAL	0 (0)	1 (0.77)
AZI-CIP-ERY-TET-NAL-CLI	7 (0.92)	12 (9.23)
AZI-CIP-GEN-TET-NAL-CLI	2 (0.26)	1 (0.77)
AZI-CIP-GEN-TET-FEN-NAL	2 (0.26)	0 (0)
AZI-CIP-TET-FEN-NAL-CLI	1 (0.13)	0 (0)
AZI-ERY-GEN-TET-NAL-CLI	1 (0.13)	0 (0)
AZI-CIP-ERY-GEN-TET	0 (0)	1 (0.77)
AZI-CIP-ERY-NAL-CLI	0 (0)	2 (1.54)
AZI-CIP-GEN-TET-NAL	3 (0.39)	0 (0)
AZI-CIP-TET-FEN-NAL	5 (0.66)	0 (0)
AZI-CIP-TET-NAL-CLI	1 (0.13)	0 (0)
CIP-ERY-GEN-TET-NAL	0 (0)	1 (0.77)
CIP-ERY-TET-NAL-CLI	1 (0.13)	0 (0)
CIP-GEN-TET-FEN-NAL	4 (0.53)	0 (0)
CIP-GEN-TET-NAL-CLI	0 (0)	1 (0.77)
CIP-TET-FEN-NAL-CLI	3 (0.39)	0 (0)
AZI-CIP-TET-NAL	6 (0.79)	2 (1.54)
CIP-ERY-TET-NAL	1 (0.13)	0 (0)
CIP-GEN-TET-NAL	29 (3.81)	14 (10.77)
CIP-TET-FEN-NAL	41 (5.39)	0 (0)
CIP-TET-NAL-CLI	4 (0.53)	1 (0.77)
CIP-ERY-TET	0 (0)	1 (0.77)
CIP-FEN-NAL	2 (0.26)	0 (0)
CIP-TET-NAL	534 (70.17)	32 (24.62)
CIP-NAL	55 (7.23)	2 (1.54)
CIP-TET	20 (2.63)	0 (0)
TET-NAL	7 (0.92)	0 (0)
CIP	3 (0.39)	0 (0)
FEN	1 (0.13)	0 (0)
NAL	1 (0.13)	0 (0)
TET	7 (0.92)	0 (0)
Susceptible	10 (1.31)	0 (0)
Total	761 (100.00)	130 (100.00)

### MLST analysis and antimicrobial resistance of erythromycin-resistant isolates

**Figure 1:**
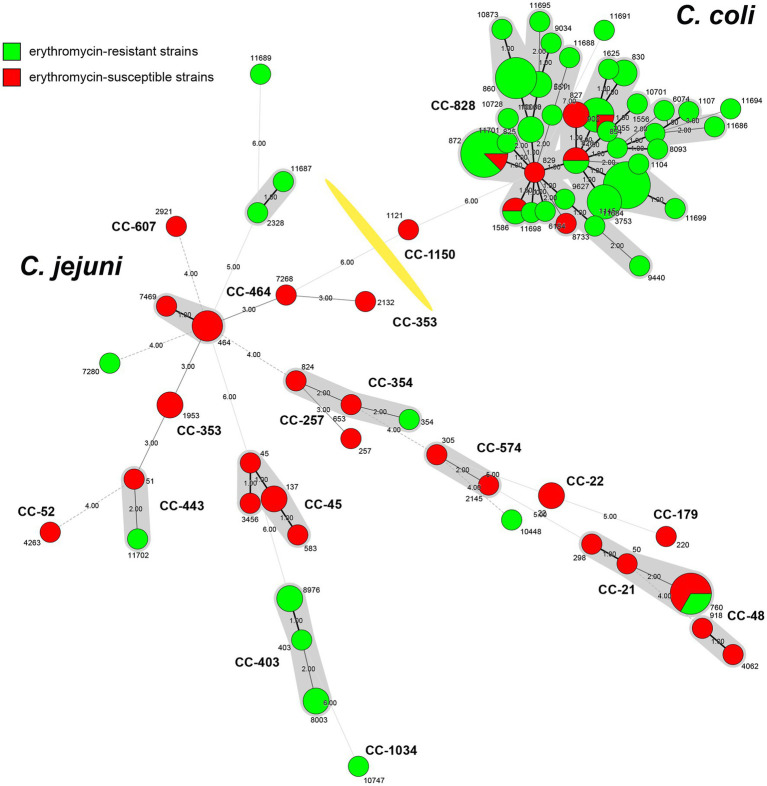
Minimum spanning tree of *Campylobacter* strains collected in Shanghai, China. Erythromycin-resistant strains from this study (*n* = 76) are indicated in green, while erythromycin-susceptible strains from unpublished data (*n* = 41) are labeled in red. Each node represents the genotypes (STs) and is proportional to the number of isolates of this ST. Clonal complexes (CCs) are illustrated around the nodes. Numbers representing allelic differences between neighboring nodes are indicated. The division of *Campylobacter jejuni* and *Campylobacter coli* is marked with a yellow division line.

According to the antimicrobial analysis, a total of 19 *C. jejuni* and 77 *C. coli* isolates resistant to erythromycin were identified. Due to the failure of recovery for 3 *C. jejuni* and 17 *C. coli* isolates from the laboratory stock, these 20 isolates were excluded from the genome extraction and further analysis. Among the 76 macrolide-resistant *Campylobacter* isolates (16 *C. jejuni* and 60 *C. coli*), a total of 47 STs were identified, including 4 novel alleles and 12 STs were newly designated in this study (Supplementary Table 1). Thirteen isolates including 6 *C. jejuni* isolates and 7 *C. coli* isolates were not assigned to any known clonal complex (CC). All 16 *C. jejuni* isolates were represented by 13 STs with a great diversity, of which 10 isolates belonged to 5 known clonal complex (CC). Minimum spanning tree (MST) analysis based on the STs profiles from the 76 *Campylobacter* isolates in this work and 41 erythromycin-susceptible strains deposited in our lab (unpublished, Supplementary Table 2) showed higher diversity of *C. jejuni* compared to *C. coli* (Figure 1). Among STs between erythromycin-resistant and susceptible stains, there were five STs in common, namely ST-760, ST-828, ST-872, ST-1055 and ST-1586.

Among the clonal complex, CC-403 was predominant (*n* = 5), followed by CC-21 (*n* = 2). The other 10 STs included one isolate, besides ST-760 of CC-21, ST-8003 and ST-8976 of CC-403 had two isolates, individually. Meanwhile, 34 STs were identified among *C. coli* isolates (*n* = 60), with the three most prevalent ST-872 (*n* = 7), ST-1145 (*n* = 8) and ST-860 (*n* = 6) were clustered into the leading CC-828 clonal complex (53, 88.33%; Figure 1). Considering the extensive high resistance to quinolones and tetracycline, all erythromycin-resistant isolates showed resistance to three or more antimicrobials, indicating that the above STs and CCs of erythromycin-resistant isolates maybe relevant to MDR of *Campylobacter.*

The most abundant pattern for *C. coli* is AZI-CIP-ERY-GEN-TET-NAL-CLI (50.00%, 30/60), including all antimicrobials except FFN. Fourteen isolates were resistant to all tested antimicrobials. For *C. jejuni*, AZI-CIP-ERY-TET-NAL-CLI was the most common resistant pattern (43.75%, 7/16), followed by AZI-CIP-ERY-GEN-TET-NAL-CLI (25.00%, 4/16). Only one *C. jejuni* isolate SH19024 of ST-10747 was resistant to all 8 antimicrobials.

### Analysis of antimicrobial resistance determinants

**Figure 2:**
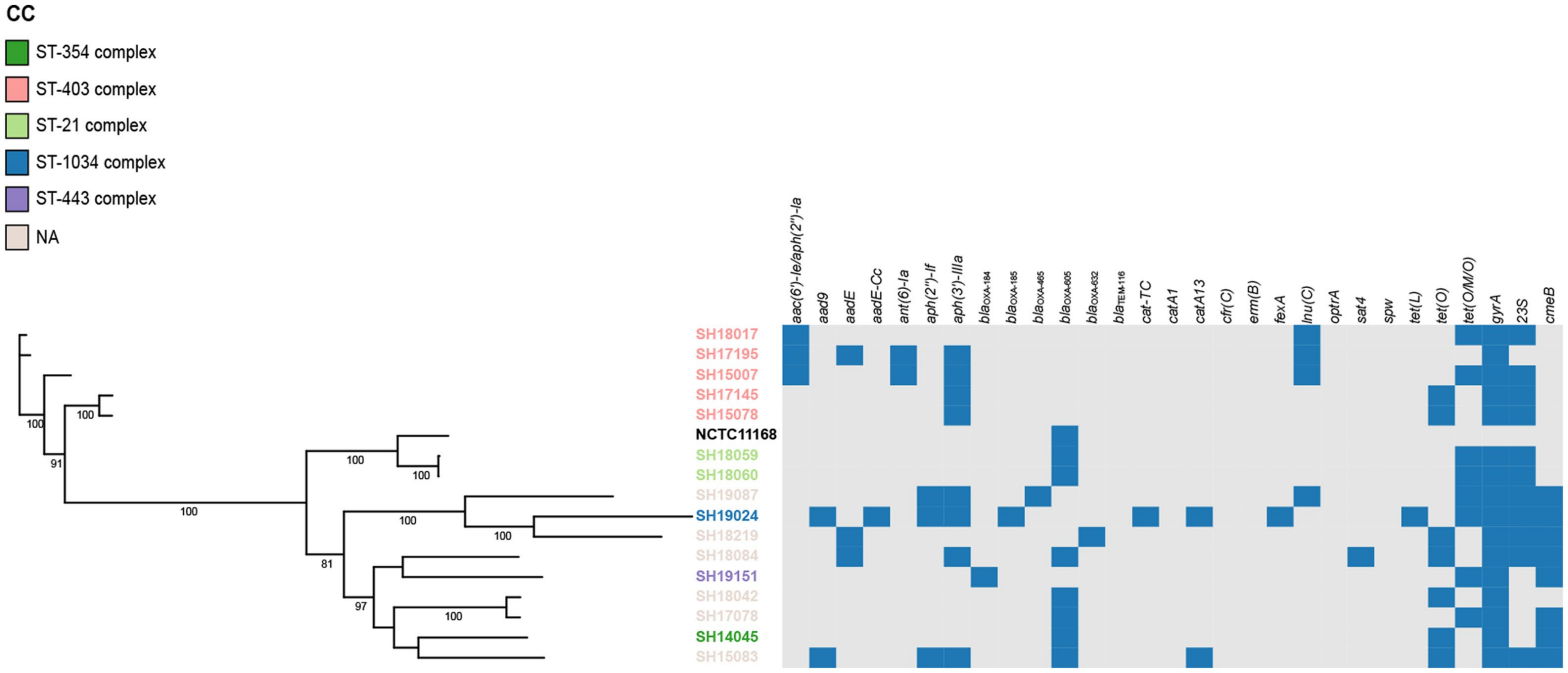
Phylogenetic analysis of erythromycin-resistant *Campylobacter jejuni* constructed by maximum likelihood using strain NCTC 11168 as reference. The isolates are featured by different color according to the corresponding clonal complexes. A heatmap of the antimicrobial resistance genes were demonstrated. The names of detected particular genes are displayed above corresponding columns. The presence of antimicrobial genes is indicated by blocks in blue. The absence of a gene is indicated by a grey block.

**Figure 3:**
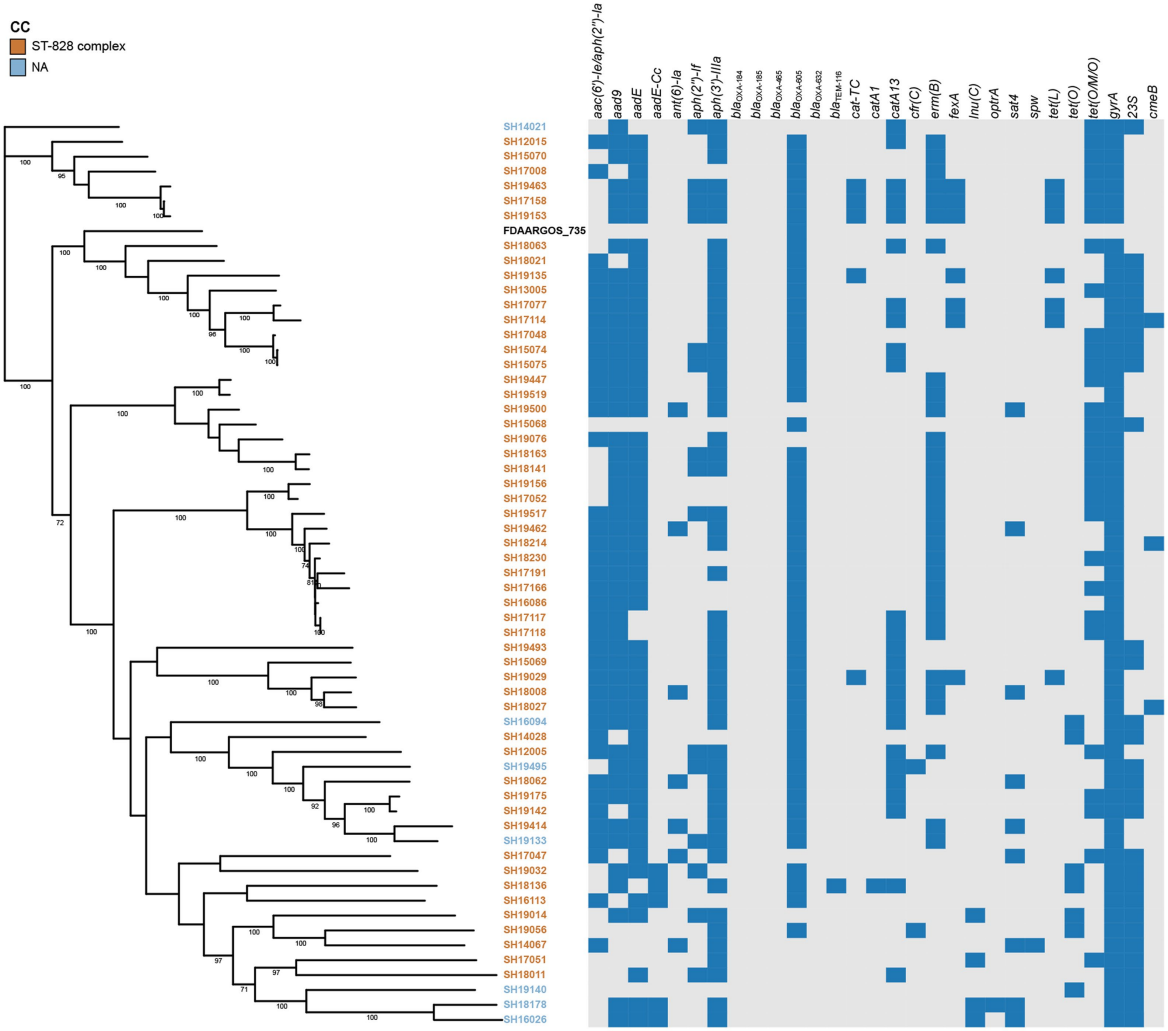
Phylogenetic analysis of erythromycin-resistant *Campylobacter coli* constructed by maximum likelihood using strain FDAARGOS_735 as reference. The isolates are featured by different color according to the corresponding clonal complexes. A heatmap of the antimicrobial resistance genes were demonstrated. The names of detected particular genes are displayed above corresponding columns. The presence of antimicrobial genes is indicated by blocks in blue. The absence of a gene is indicated by a grey block.

The presence of resistance genes was determined based on the genome contigs of 76 erythromycin-resistant isolates (16 *C. jejuni* and 60 *C. coli*), detailed in Table 3. *In silico* analysis revealed that point mutations in the *gyrA* gene (T86I), confering resistance to quinolone (Griggs et al., 2005), was present in all isolates, which may account for the extremely high resistance to CIP and/or NAL. The erythromycin and lincosamide resistance associated mutations in the 23S rRNA gene at 2074 and 2075 positions were analyzed, and A2075G transition was the only mutation identified in 30 *C. coli* (50.00%) and 11 *C. jejuni* isolates (68.75%). CmeABC is a prevalent multi-drug efflux pump system broadly distributed in *Campylobacter*, representing an important mechanism for antibiotic resistance (Lin et al., 2002). The contribution of the CmeABC system has been studied in conferring intrinsic resistance to antibiotics erythromycin, tetracycline, ciprofloxacin and a range of other antimicrobial agents (Lin et al., 2002; Sharifi et al., 2021). It consists of three components: a periplasmic fusion protein CmeA, an inner membrane drug transporter CmeB, and an outer membrane protein CmeC (Lin et al., 2002). Variant *cmeB* sequence of the resistance-enhancing variant (RE-CmeABC) derived from *C. coli* DH161 (Yao et al., 2016) have been detected in 8 *C. jejuni* (50.00%) and 3 *C. coli* (5.00%) isolates, with a higher proportion in *C. jejuni* than in *C. coli* isolates (Table 3). As illustrated in Figures 2, 3, isolates carrying RE-*cmeB* were distributed in diverse clusters, suggesting *cmeB* might be horizontal transferable among *Campylobacter* strains.

**Table 3:** Antimicrobial resistance profiles detected in 76 erythromycin-resistant *Campylobacter* isolates.

	Resistance genes	*Campylobacter jejuni* (*n* = 16)		*Campylobacter coli* (*n* = 60)		*χ* ^2^	*P*
		Presence	%	Presence	%		
Quinolones	*gyrA (T86I)*	16	100.00	60	100.00		
Macrolides	*23S rRNA (A2075G)*	11	68.75	30	50.00	1.787	0.181
	*erm(B)*	-	-	30	50.00		
Multidrug Efflux pump	*RE-CmeB*	8	50.00	3	5.00	17.188	0
Aminoglycosides	*aac(6*′*)-Ie/aph(2*″*)-Ia*	3	18.75	39	65.00	10.929	0.001
	*aph(2*″*)-If*	3	18.75	15	25.00	0.037	0.848
	*aad9*	2	12.50	48	80.00	25.571	0.000
	*aadE*	3	18.75	51	85.00	23.830	0.000
	*aadE-Cc*	1	6.25	5	8.33		1.000
	*ant(6)-Ia*	2	12.50	7	11.67	0.000	1.000
	*aph(3*′*)-IIIa*	8	50.00	49	81.67	5.172	0.023
	*sat4*	1	6.25	9	15.00	0.254	0.614
	*spw*	-	-	1	1.67		
Beta-lactams	*bla*_OXA-61_ *family*	7	43.75	49	81.67	7.512	0.006
	*bla*_OXA-184_ *family*	4	25.00	-	-		
	*bla*_TEM-116_	-	-	1	1.67		
Phenicols	*cat-TC*	1	6.25	5	8.33		1.000
	*catA1*	-	-	1	1.67		
	*catA13*	2	12.50	25	41.67	4.691	0.030
	*fexA*	1	6.25	7	11.67	0.029	0.866
	*cfr(C)*	-	-	2	3.33		
	*optrA*	-	-	1	1.67		
Lincosamides	*lnu(C)*	4	25.00	4	6.67	2.771	0.096
Tetracyclines	*tet(L)*	1	6.25	7	11.67	0.029	0.866
	*tet(O)*	7	43.75	7	11.67	6.649	0.010
	*tet(O/M/O)*	8	50.00	30	50.00	0.000	1.000

Several genes related to aminoglycoside resistance appeared commonly in both *C. coli* and *C. jejuni* isolates (Table 3). Of these genes, *aac(6*′*)-Ie/aph(2*″*)-Ia* (*n* = 42), *aad9* (*n* = 50), *aadE* (*n* = 54) and *aph(3*′*)-IIIa* (*n* = 57) were more frequently detected in *C. coli* than in *C. jejuni* (*aac(6*′*)-Ie/aph(2*″*)-Ia*, *χ*^2^ = 10.929, *p* = 0.001; *aad9*, *χ*^2^ = 25.571, *p* < 0.001; *aadE*, *χ*^2^ = 23.830, *p* < 0.001; *aph(3*′*)-IIIa*, *χ*^2^ = 5.172, *p* = 0.023). The presence of *bla*_OXA-605_ encoding for beta-lactamase of OXA-61 family, was identified in 56 isolates comprising of *C. coli* (*n* = 49) and *C. jejuni* (*n* = 7), while that of OXA-184 family (including *bla*_OXA-184_, *bla*_OXA-185,_ *bla*_OXA-465_, *bla*_OXA-632_) existed only in 4 *C. jejuni* isolates, and zero *C. coli* isolates. Fourteen isolates harbored tetracycline resistance genes *tet*(O), while 38 isolates carried mosaic form *tet*(O/M/O) (Table 3). In addition, the prevalent gene associating with phenicol resistance *catA13* was detected in 27 isolates. Thirty *erm*(B)-positive *C. coli* isolates were isolated, while no *erm*(B)-positive *C. jejuni* were identified (Table 3). The profile of the resistance genes described above, as well as other resistant determinant for streptothricin, beta-lactam antibiotics, chloramphenicol, and lincosamide were also illustrated in Figures 2, 3.

### Phylogenetic analysis of erythromycin-resistant isolates

On the basis of recombination-free core SNPs, phylogenetic analysis was performed on erythromycin-resistant 16 *C. jujuni* and 60 *C. coli* isolates illustrated in Figures 2, 3, respectively. Overall, most strains from the same CC or with little allelic variation were clustered in closely related branches. It is shown that SH19463/SH17158/SH19153 (Figure 3), SH17145/SH15078 and SH18059/SH18060 (Figure 2) share the same resistance patterns. Specifically, SH18059 and SH18060 were isolated successively from patients with diarrhea caused by foodborne illness in the same district in Shanghai, indicating an epidemiological association. Among these isolates, phylogeny of *C. coli* showed less diversity than *C. jejuni*, with most strains clustered to ST-828 clonal complex (Figures 2, 3). Additionally, *erm*(B) was detected exclusively in *C. coli* isolates with 13 STs belonging to the predominant ST-828 clonal complex except for SH19133 of ST-9034, which is not belong to any known clonal complex (Figure 3). All ST1145 strains (*n* = 8) in our study harbored *erm*(B), demonstrating this genotype maybe associated with the dissemination of *erm*(B), which require further analysis (Supplementary Table 2, Figure 3).

**Figure 4:**
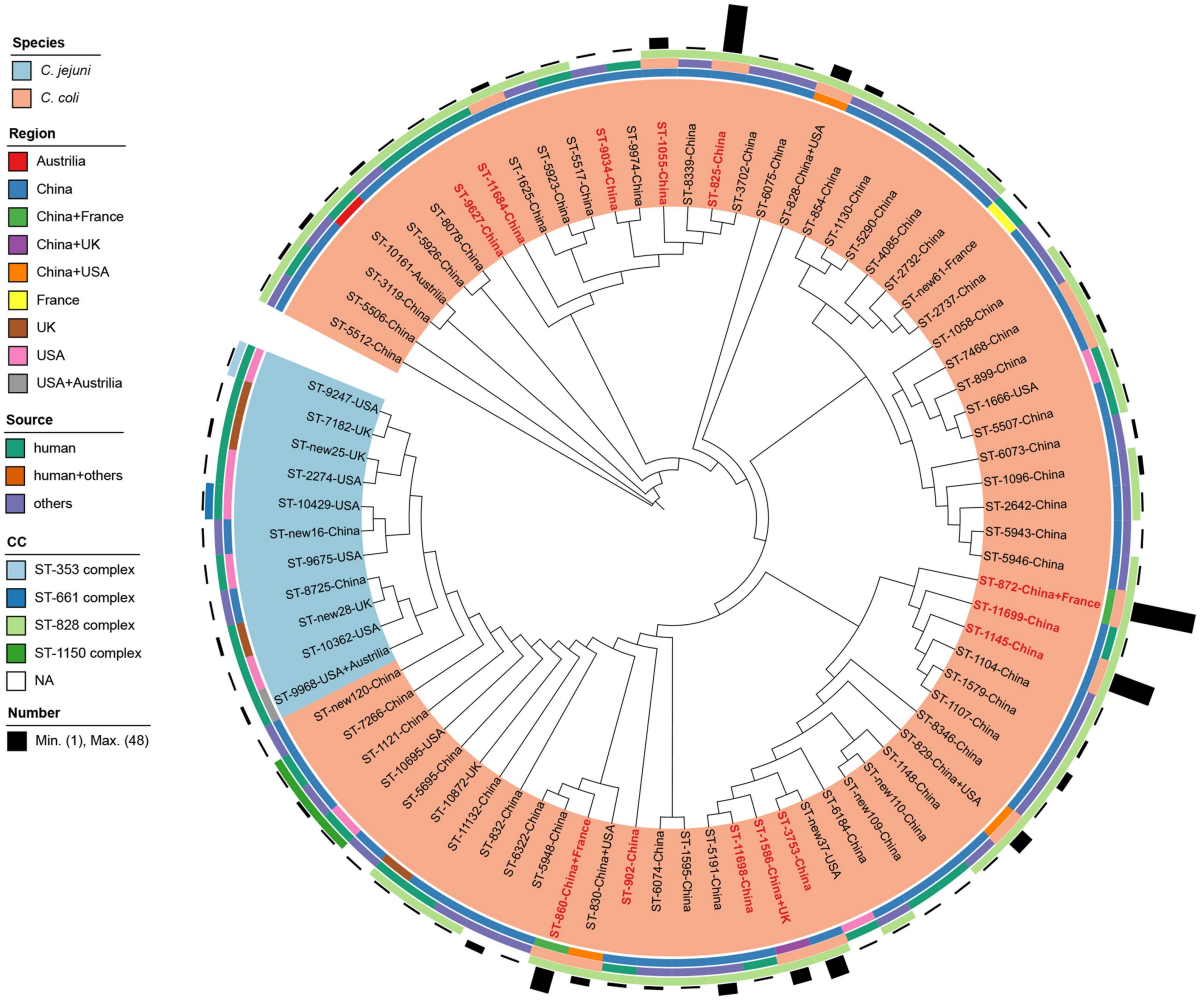
Comparison of *erm*(B)-carrying isolates identified in this study, reported previously and deposited in National Database of Antibiotic Resistant Organisms. Isolates are indicated with font labels referring to ST with the country of isolate regions, and shaded by different color according to the species. Sequence types identified in this study are colored in red. The four outer illustrations including three rings and one bar chart from inside to outside show the isolation regions, sources, clonal complexes and corresponding number of *erm*(B)-carrying *Campylobacter* strains.

### Comparison of *erm*(B)-carrying isolates

A total of 30 *erm*(B)-carrying *C. coli* isolates were identified in this study. No *erm*(B) gene was detected in *C. jejuni* in our study. All these *erm*(B) positive isolates were multi-drug resistant. The most prevalent resistant pattern was AZI-CIP-ERY-GEN-TET-NAL-CLI (60.00%, 18/30). Seven isolates were resistant to all tested antimicrobials, while five isolates show resistance to all antimicrobials except gentamicin and florfenicol. As shown in Figure 4, all *erm*(B)-harboring *C. coli* isolates from patients with diarrhea in Shanghai were selected for comparison with 264 *erm*(B)-positive *Campylobacter* isolates reported previously (Wang et al., 2014; Liu et al., 2017, 2019; Wallace et al., 2020) and isolates available in National Database of Antibiotic Resistant Organisms, including 14 *C. jejuni* and 280 *C. coli* isolates together. The information of the selected isolates is shown in Supplementary Table 3. A total of 79 STs were recovered from the 294 isolates, with eight unassigned new ST, of which 57 STs were classified to four known CCs, namely CC-828 (*n* = 258), CC-1150 (*n* = 4) for *C. coli* and CC-353 (*n* = 1), CC-661 (*n* = 1) for *C. jejuni*, respectively. *C. coli* isolates belonging to CC-828 comprised strains from five countries, including China (*n* = 246), France (*n* = 2), the United States (*n* = 4), the United Kingdom (*n* = 5), and Australia (*n* = 1) (Figure 4). Cluster CC-1150 was composed of 4 *C. coli* isolates sampled from clinical and chicken feces from the Unites States and China. Cluster CC-353 and CC-661 were constituted by ST-9247 and ST-10429 of one *C. jejuni* isolate from clinical source in the United States, respectively. Among these isolates, all 14 *C. jejuni* isolates were clustered to 11 STs, with three unassigned new STs, of which ST-9247 and ST-10429 were classified to CC-353, CC-661, respectively. The remaining STs of *C. jejuni* isolates did not belong to any known CC. The majority of *C. jejuni* isolates (92.86%, 13/14) were sampled from humans in China, the United States, the United Kingdom, and Australia. Whereas 280 *C. coli* isolates were clustered to 63 STs and 5 new STs, with ST-872 being predominant (17.14%, 48/280), followed by ST-825 (12.86%, 36/280) and ST-1145 (11.43%, 32/280). There were 13 STs (16.46%, 13/79) found in poultry, livestock, as well as human disease cases. Among the 13 STs identified in this study, ST-1145 (26.67%, 8/30) dominated, followed by ST-872 (20%, 6/30) and ST-3753 (13.33%, 4/30), and ST-9034, ST-9627, ST-11684, ST-11698, ST-11699 were reported as *erm*(B) harboring isolates for the first time.

## Discussion

*Campylobacter* remains one of the major causes of gastroenteritis worldwide, with the increasing incidence and prevalence of *Campylobacter* infection in both developed and developing countries (Kaakoush et al., 2015). The present study provides information on the phenotypic resistance of clinical isolates collected during 2012–2019 in Shanghai, China, as well as prevalence of isolates resistant to the macrolide (erythromycin in this case), its underlying genetic determinants and phylogenetic relationships with the whole genome sequencing (WGS) data. Several studies have demonstrated the antimicrobial resistance prevalence and genomic characterization of *Campylobacter* from clinical samples (Zhou et al., 2016; Yu et al., 2020; Zhang et al., 2020), poultry (Liu et al., 2017), livestock (Li et al., 2022) and wild birds samples (Du et al., 2019) in China. Data covering the 8 years showed that clinical *Campylobacter* isolates in Shanghai exhibited a continuous extremely high-level resistance to ciprofloxacin, nalidixic acid and tetracycline, with a similar trend in Beijing (Zhou et al., 2016; Zhang et al., 2020), Shanghai (Du et al., 2018), and Peru (Quino et al., 2022) from human isolates in previous studies. *Campylobacter jejuni* and *C. coli* showed 96.5 and 100% of resistance to ciprofloxacin, which is higher than the occurrence in European Union (EU), with ciprofloxacin resistance of 61.2 and 65.8% for *C. jejuni* and *C. coli*, respectively (Authority, 2022). *Campylobacter* resistance to erythromycin in this study (2.50% for *C. jejuni* and 59.23% for *C. coli*) is similar to Beijing (Zhang et al., 2020) and Peru (Quino et al., 2022), but higher than that in EU (0.7% for *C. jejuni* and 10% for *C. coli*; Authority, 2022). The level of erythromycin resistance was higher in *C. coli* than in *C. jejuni,* in accordance with other studies (Florez-Cuadrado et al., 2017; Authority, 2022; Liao et al., 2022; Quino et al., 2022). The proportions of combined resistance to both ciprofloxacin and erythromycin were 2.37% (18/761) and 59.23% (77/130) for *C. jejuni* and *C. coli,* which were higher than 0.5 and 9.0% in *C. jejuni* and *C. coli* isolates at EU (Authority, 2022). The recent reported antibiotic resistance among *C. coli* and *C. jejuni* isolates varied with respect to the country of isolation may be related to the source of isolation, the antimicrobial usage in both animal agriculture and human medicine in different geographic regions, probably by enhancing the selection pressure for antibiotic-resistant isolates (Gibreel and Taylor, 2006; Moore et al., 2006). The widely observed higher rate of macrolide resistance in *C. coli* than in *C. jejuni* may be associated with fitness costs impacts of certain antibiotic-resistant mutants, with the underlying mechanisms remain to be further elucidated (Bolinger and Kathariou, 2017). Since ciprofloxacin and erythromycin are important for treatment of campylobacteriosis, the resistance level could potentially relate to therapeutic failure of antibiotic treatment, considering the extremely high quinolone resistance (Engberg et al., 2001; Dai et al., 2020).

Further genetic analyses of 76 isolates resistant to erythromycin revealed high genetic diversity of isolates in Shanghai, while *C. coli* isolates showed less diversity overall as well as previous study (Sheppard et al., 2012). We determined 47 STs, with 12 STs were new designated. The predominant sequence type ST-872 (8 isolates), ST-1145 (8 isolates), ST-860 (6 isolates), ST-3753 (4 isolates) accounted for one third of the erythromycin-resistant *C. coli* isolates. The STs except for ST-3753 have been reported previously as primary STs of erythromycin-resistant *C. coli* isolates, while major STs of *C. jejuni* were distinct from our study, confirming the genotype diversity of *C. jujuni* (Zhang et al., 2016).

The most prevalent clonal complexes were CC-403 (*n* = 5) and CC-21 (*n* = 2) for *C. jejuni* and CC-828 (*n* = 55) for *C. coli*, which were frequently isolated in China (Ju et al., 2018; Zhang et al., 2020). CC-21 and CC-828 are known host generalist lineages containing the largest number of isolates from multiple sources and associated with human campylobacteriosis worldwide, representing 18.02 and 15.50% isolates according to the *C. jejuni/coli* isolates database updated at Feb 22, 2023 (Jolley et al., 2018). The ST-403 complex was reported as the clonal genotype associated with a neuropathogenic *C. jejuni* serotype among 49 *C. jejuni* isolates derived from patients with enteritis or GBS (Islam et al., 2009), and demonstrating strong antimicrobial resistance (Ju et al., 2018). CC-828 and CC-403 have been demonstrated related to MDR of *Campylobacter* spp., previously (Zhang et al., 2020). In the present study, five isolates belonging to ST-403 complex displayed multidrug resistance to six to seven antibiotics. CC-403 isolates represent 0.97% of the database, most of which were from human clinical cases, pigs and cattle, seldom isolated from poultry, indicating that the complex may have evolved to become less suited to colonization of the avian host (Morley et al., 2015) and disseminated to human through livestock avenue. Highly diverse erythromycin-resistant isolates in this study may have the capacity to cause human disease, with the potential of spreading among different reservoir hosts.

By detecting the homologous sequence of *cmeB* variant gene in *C. coli* DH161, our study revealed that RE-*CmeABC*-positive isolates were genetic diverse, suggesting that horizontal gene transfer other than clonal expansion was involved in the spread of the CmeABC variant among isolates derived from Shanghai. Both overall genetic diversity and regional clonality were observed previously with the former one dominated (Yao et al., 2016), this difference might be attribute to the limitation of sampling number and source host. The prevalence of RE-*cmeABC* in *C. jejuni* was higher in our study, concordance with the perception that it confers an adaptation advantage under antimicrobial selection pressure as *C. jejuni* is intrinsically less resistant to antimicrobials (Wang et al., 2015). This finding demonstrated that RE-*cmeABC* could be horizontally transferable more rapidly in *C. jejuni*.

Macrolide resistance in *Campylobacter* species can be mainly attributed to the *erm*(B) and A2075G mutation with the latter one dominated (Gibreel and Taylor, 2006; Payot et al., 2006). *erm*(B) has been commonly detected in China since the first discovery in 2014 (Qin et al., 2014; Wang et al., 2014; Zhang et al., 2016; Liu et al., 2017, 2019), conferring high-level resistance to macrolide-lincosamide-streptogramin B. In this study, among the 76 erythromycin-resistant isolates, the presence of *erm*(B) in our study (39.5%, 30/76) was higher than that in previous work (Wang et al., 2014; Zhang et al., 2016), in which 18.4% of erythromycin-resistant isolates harbored the *erm*(B) gene. While A2075G mutation in 23S rRNA was the main underlying mechanism of erythromycin resistance, with 41 isolates (53.9%) had the mutation, which is lower than 83% reported in previous study (Wang et al., 2014; Zhang et al., 2016). The A2075G mutation and the *erm*(B) gene accounted for majority of the erythromycin resistance, with neither was detected in five erythromycin resistant isolates. Despite the report of coexistence of the *erm*(B) and the A2075G mutation in previous studies (Wang et al., 2014; Zhang et al., 2016), our study revealed that no isolates carried both the *erm*(B) and the A2075G mutation simultaneously obtained from diarrheal cases. Previous study demonstrated that the difference may be attributed to the different isolation source (Zhang et al., 2016). As two main macrolide resistance determinants, given the fitness loss incurred by the 23S rRNA mutation and the horizontally transferable of *erm*(B), the prevalence of these two mechanisms in our study indicated that the emergency and spread of *erm*(B) may provide an alarming threat to the antimicrobial treatment of severe campylobacteriosis. It should be noted that nine out of 10 human *erm*(B)-positive isolates in previous study were from Shanghai, implying more data for a better overview of the situation of *erm*(B) dissemination among humans in this district were needed.

By comparing of *erm*(B)-carrying *Campylobacter* isolates in our study with *erm*(B)-carrying isolates worldwide, seven STs out of 13 STs in our study were found of multiple source attributions, indicating *erm*(B) are at risk of zoonotic transmission to humans *via* natural transformation. The majority of ST-872 (6/8) and all ST-1145, ST-3753 isolates carried the *erm*(B) gene. Similarly, Zhang et al. reported that all eight ST-1145 isolates from diarrheal patients and chicken carried the *erm*(B) gene (Zhang et al., 2016). A study of *erm*(B) incidence in *Campylobacter* isolates from both human diarrheal cases and food-producing animals showed that ST-1145 were prevalent *erm*(B)-positive isolates from diarrheal patients in Shanghai (Wang et al., 2014), suggesting the *erm*(B) gene may be somewhat related to the ST-1145 clone which need further analysis.

In summary, our study revealed the antimicrobial resistances of *Campylobacter* isolates from outpatients with diarrhea in Shanghai between 2012 and 2019 and indicated that the isolates are highly resistant, especially to quinolones and tetracyclines among both *C. jejuni* and *C. coli* strains. Detailed information about phylogeny, genotypes and distribution of resistance determinant of erythromycin resistant *Campylobacter* spp. based on genomic data were provided. Importantly, the prevalence of erythromycin-resistant isolates and *erm*(B) gene pose threats to public health. Sustained surveillance is necessary to monitor the resistance profiles of *Campylobacter* stains with macrolide resistance and to estimate the potency of strategies focused on the control of antimicrobial resistance.

## Data availability statement

The datasets presented in this study can be found in online repositories. The names of the repository/repositories and accession number(s) can be found below: https://www.ncbi.nlm.nih.gov/, PRJNA911465.

## Ethics statement

The protocols were approved by the Ethics Committee of Shanghai Municipal Center for Disease Control and Prevention (Shanghai CDC). Clinical specimens were collected from the routine surveillance program for patients with diarrhea of Shanghai CDC.

## Author contributions

FG, LT, XZ, and MinC conceived the study and revised the manuscript. FG performed the experimental work, analyzed the data, and drafted the manuscript. MingC and HC contributed to the analysis and interpretation of data. YZ and JL were involved in the antibiotic susceptibility analysis. All authors contributed to the article and approved the submitted form.

## Funding

This work was sponsored by Shanghai Sailing Program (No. 21YF1439200) and Shanghai Outstanding Youth Training Program of the Three-Year Public Health Action Plan (No. GWV-10.2-YQ07).

## Conflict of interest

The authors declare that the research was conducted in the absence of any commercial or financial relationships that could be construed as a potential conflict of interest.

## Publisher’s note

All claims expressed in this article are solely those of the authors and do not necessarily represent those of their affiliated organizations, or those of the publisher, the editors and the reviewers. Any product that may be evaluated in this article, or claim that may be made by its manufacturer, is not guaranteed or endorsed by the publisher.

## Footnotes

^1^http://pubmlst.org/campylobacter/

^2^https://github.com/tseemann/abricate

^3^https://www.ncbi.nlm.nih.gov/pathogens/antimicrobial-resistance/AMRFinder/
