# Supplementary material for: Erythromycin resistance of clinical Campylobacter jejuni and Campylobacter coli in Shanghai, China
Source: Front Microbiol. 2023 May 16;14:1145581. doi: 10.3389/fmicb.2023.1145581 (PMC10229067; doi:10.3389/fmicb.2023.1145581)
Supplement: Supplementary file 1 [file Table_1.DOCX]

Supplementary Table S1. New STs and alleles of 76 *Campylobacter* isolates from Shanghai

| Isolates | aspA | glnA | gltA | glyA | pgm | tkt | uncA | ST | Clonal complex | PubMLST database id |
| --- | --- | --- | --- | --- | --- | --- | --- | --- | --- | --- |
| SHCDCCB12005 | 33 | 39 | 30 | 82 | 112 | 43 | 139 | 11684 | ST-828 complex | 111609 |
| SHCDCCB12015 | 33 | **807^#^** | 30 | 82 | 113 | 43 | 17 | 11698 | ST-828 complex | 111619 |
| SHCDCCB16026 | 33 | 38 | 473 | 82 | 104 | 35 | 36 | 11686 |  | 111610 |
| SHCDCCB16086 | 33 | 39 | **686** | 82 | 104 | 44 | 17 | 11699 | ST-828 complex | 111620 |
| SHCDCCB18021 | 33 | **808** | 30 | 82 | 113 | 43 | 17 | 11701 | ST-828 complex | 111621 |
| SHCDCCB18042 | 8 | 2 | 2 | 212 | 350 | 253 | 147 | 11687 |  | 111611 |
| SHCDCCB18178 | 33 | 38 | 30 | 82 | 113 | 35 | 68 | 11688 |  | 111612 |
| SHCDCCB18219 | 8 | 364 | 80 | 470 | 470 | 772 | 533 | 11689 |  | 111613 |
| SHCDCCB19087 | 22 | 490 | 292 | 26 | 127 | 24 | 412 | 11691 |  | 111614 |
| SHCDCCB19140 | 33 | 38 | 44 | 79 | 113 | 43 | 36 | 11694 |  | 111615 |
| SHCDCCB19151 | 7 | **809** | 2 | 15 | 11 | 3 | 12 | 11702 | ST-443 complex | 111622 |
| SHCDCCB19495 | 33 | 38 | 30 | 82 | 112 | 47 | 139 | 11695 |  | 111616 |

SHCDCCB18042, SHCDCCB18219, SHCDCCB19087 and SHCDCCB19151 are *C. jejuni,* the others are *C. coli*.

^#^New alleles are labeled in bold.
